# Association between Hypoadiponectinemia and Low Serum Concentrations of Calcium and Vitamin D in Women with Polycystic Ovary Syndrome

**DOI:** 10.5402/2012/949427

**Published:** 2012-01-16

**Authors:** Sahar Mazloomi, Faranak Sharifi, Reza Hajihosseini, Sadroddin Kalantari, Saideh Mazloomzadeh

**Affiliations:** ^1^Clinical Biochemistry, Metabolic Diseases Research Center, Zajan University of Medical Sciences, 451398-6745 Zanjan, Iran; ^2^Clinical Endocrinology, Metabolic Diseases Research Center, Zajan University of Medical Sciences, 451398-6745 Zanjan, Iran; ^3^Department of Biochemistry, Payame Noor University, Tehran, Iran; ^4^Biochemistry, Zanjan University of Medical Sciences, Zanjan, Iran; ^5^Epidemiology, Zanjan Metabolic Diseases Research Center, Zanjan, Iran

## Abstract

*Objective*. To investigate the possible association of calcium and vitamin D deficiency with hypoadiponectinemia in women with PCOS. *Subjects and Methods*. In this case-control study, 103 PCOS cases and 103 controls included. The concentrations of calcium, 25-OH-vitamin D (25OHD), adiponectin, insulin, glucose, total cholesterol, HDL-cholesterol, triglyceride (TG), and androgens were measured in fasting blood samples. *Results*. Adiponectin (8.4 ± 2.7 ng/mL versus 13.6 ± 5 ng/mL in control group, *P* : 0.00), calcium (2 ± 0.1 mmol/L versus 2.55 ± 0.17 mmol/L in controls, *P* : 0.00), and 25-OH-Vit D (30 ± 2.99 nmol/L versus 43.7 ± 5.2 nmol/L in control group, *P* : 0.00) levels were decreased in women with PCOS. Subjects with PCOS had higher concentrations of TG (1.4 ± 0.77 mmol/L versus 1.18 ± 0.75 mmol/L in control group, *P* : 0.019) and dehydroepiandrosterone sulfate (DHEA-S) (10.7 ± 11 mmol/L versus 9.7 ± 10.4, *P* : 0.02 in control group). There were significant correlations between adiponectin concentrations with calcium (*r* : 0.78, *P* : 0.00) and 25OHD levels (*r* : 0.82, *P* : 0.00). The association of hypoadiponectinemia and PCOS was not significant considering 25OHD as a confounding factor. *Conclusion*. The present findings indicate that the association of hypoadiponectinemia with PCOS is dependent on vitamin D. A possible beneficiary effect of vitamin D on the metabolic parameters in PCOS may be suggested.

## 1. Introduction

Obesity, insulin resistance, and type 2 diabetes are increasingly prevalent worldwide [1], and adipocyte secretory proteins (adipokines) are associated with the development of insulin resistance. Adiponectin is a major adipokine [2], and low levels of adiponectin are linked to diabetes, insulin resistance, coronary heart disease, and metabolic syndrome [3, 4].

On the other hand, alterations in vitamin D metabolism and parathyroid hormone (PTH) concentrations are closely related to obesity as well as the clinical aspects of the metabolic syndrome. Despite a larger total surface area to expose to sunlight to produce more vitamin D in obesity, some studies have revealed decreased concentrations of 25OHD in obesity [5–7]. Lower mobility and consequently less exposure to solar ultraviolet radiation may be a possible explanation for the increased risk of relative vitamin D deficiency in obesity [8].

Calcium ions engage in regulation of various physiological processes including muscle contraction, cell adhesion, cell division and growth, ion transport, protein folding, protein degradation, gene transcription, apoptosis, and exocytosis [9]. In addition, a number of proteins, such as members of the S100 family of calcium binding proteins, are known to form higher order oligomers in a calcium-dependent manner [10, 11].

Adiponectin isoforms secreted from adipose tissue and adipocytes following calcium chelation or addition have been investigated. In all cases, calcium chelators reduced the level of HMW isoforms, while the addition of calcium increased formation of HMW adiponectin [12].

Polycystic ovary syndrome (PCOS) is characterized by chronic anovulation and hyperandrogenism [9–11]. PCOS can be considered as the most frequent cause of anovulation [13] and is a prevalent endocrine disorder among women [14]. Insulin resistance with compensatory hyperinsulinaemia and low adiponectin concentrations independent of BMI has been reported in PCOS [15]. However, calcium and vitamin D concentrations in women with PCOS and their relationship with adiponectin have not been thoroughly studied. We therefore designed the present study to investigate (a) the relationship between serum 25OHD, calcium, and adiponectin concentrations in PCOS and (b) the possible associations of the above elements with the hormonal and metabolic characteristics of the syndrome.

## 2. Subjects and Methods

### 2.1. Subjects

A total of 206 women, aged between 15 and 40 years, including 103 newly diagnosed PCOS cases and 103 healthy controls were enrolled serially in the study. All the patients were outpatients at the Endocrine Clinic of Vali-e-asr Hospital of Zanjan University of medical sciences and diagnosed as PCOS based on the Rotterdam criteria [16]. All the subjects with secondary causes of PCOS such as prolactinoma, congenital adrenal hyperplasia, cushing syndrome, and virilizing ovarian or adrenal tumors were excluded. Control subjects were from the same socioeconomic population who were matched for their age and body mass index (BMI) with the cases. All women in control group had normal ovulating cycles and no signs of hyperandrogenism. None of the women had either any systemic disease or use of any medication that might affect their reproductive physiology.

 Approval was obtained from the ethics committee of Zanjan University of Medical Sciences. All the participants were notified about the goals of the study, and informed consent was obtained. There was no cost to the participants for the biochemical tests, and the results of these measures were reported to them.

### 2.2. Measurements

Body weight was measured to the nearest 0.1 kg with a balanced-beam scale while wearing light clothing, and height was measured with a stadiometer to the nearest 0.5 cm. BMI was calculated based on the weight/(height)² formula. Waist circumferences between the lowest rib and the iliac crest, at the level of umbilicus, were measured in duplicate to the nearest millimeter using flexible tape. 

In all the women, blood samples were collected between days 3 and 6 of a spontaneous menstrual cycle, at 08-09 Am, after an at least 12 hours fast. The basal levels of LH, FSH, testosterone, dehydroepiandrosterone sulfate(DHEA-S), adiponectin, Insulin, plasma glucose, total calcium, 25OHD and lipid profile were measured. Homeostasis model assessment index (HOMA Index) was used to determine the level of insulin resistance and was calculated according to the equation [17]:

(1) [Insulin  (μU/mL)][FPG  (mmol/L)]/22.5.

Insulin resistance was considered as HOMA index more than 2.1 [18]. Insulin, LH, FSH, and testosterone levels were measured with electrochemiluminescence immunoassay (ECLIA) using commercial kits (DRG, Germany) and adiponectin with ELISA using highly sensitive kits (Biovendor, Germany, no. RD191023100). The sensitivity of the assay was 0.5 ng/mL, and its assay range was 1–150 ng/mL. Serum calcium levels were determined by spectrophotometer, and 25OHD was measured using ELISA (DRG, Germany). The sensitivity of the assay was 2 nmol/L, and its assay range was 6.4–250 nmol/L. Vitamin D deficiency was defined as a serum level of 25OHD of ≤30 nmol/L and insufficiency as a serum level between 30 and 75 nmol/L.

### 2.3. Statistical Analysis

Data are presented as mean ± SD. Statistical analysis was conducted using SPSS version 11.5. Proportions were compared by using the Chi-square test. Group means were compared using the Student's *t-*test and Mann-Whitney test. The Pearson correlation statistic was used to investigate correlations between variables. Bivariate correlation analysis (calculation of the Pearson coefficient) was used to assess the correlation of serum adiponectin, calcium, and vitamin D levels to each parameter. Multiple logistic regression analyses were used to assess the independent effect of hypovitaminosis D and low calcium levels on the odds for PCOS after adjustment for confounding factors. Independent relationships between serum adiponectin levels and those parameters to which they were found to correlate significantly were assessed using multiple linear regression models. Statistical significance was set at *P* < 0.05.

## 3. Results

The anthropometric, hormonal, and metabolic features of the women are studied, and their statistical significance are summarized in Table 1.

Although PCOS was associated with higher DHEA-S concentrations, no significant difference was seen for testosterone concentrations between the two groups. LH was significantly higher in the subjects with PCOS.

Table 2 shows the mean value for calcium, vitamin D, and adiponectin in the two groups. Subjects with PCOS had lower calcium and 25OHD concentrations. Using logistic regression analysis, lower concentration of calcium and vitamin D can be seen in PCOS even after adjustment for other confounding factors (Tables  3(a) and 3(b)).

Furthermore PCOS had a significant negative effect on adiponectin concentrations independent of BMI and waist circumference. The risk of having PCOS was significantly higher for those with lower concentrations of adiponectin (OR: 0.6, *P* < 0.001). This risk estimation remained significantly positive after adjustment for calcium (OR: 2.3, *P* : 0.04). After adjustment for vitamin D concentration, no association was found between hypoadiponectinemia and PCOS (Table 4).

A significant negative correlation was seen in the participants between adiponectin and insulin (*r* : −0.24, *P* : 0.009) even after adjustment for BMI. Adiponectin was correlate potently to calcium (*r* : 0.8, *P* < 0.001) and 25OHD (*r* : 0.83, *P* < 0.001) in the both groups of the participants. Thirty-eight people of the participants (21.6%) are recognized to have vitamin D deficiency, and 99 subjects (56%) had vitamin D insufficiency. In comparison to those with vitamin-D sufficient status, these people had higher waist circumference (87 ± 12 cm versus 82.3 ± 13.7 cm resp., *P* : 0.04), BMI (29 ± 4.5 kg/m^2^ versus 26.5 ± 5 kg/m^2^ resp., *P* : 0.008), and lower adiponectin concentration (6.7 ± 2.3 ng/mL versus 12 ± 4.4 ng/mL resp., *P* < 0.001).

Although there was a significant negative correlation between insulin, calcium (*r* : −0.29,  *P* : 0.001), and 25OHD (*r* : −.29, *P* : 002), these correlations were not significant after adjustment for BMI and waist circumference (Table 5).

There was a significant correlation between triglyceride concentration and calcium (*r* : −0.2, *P* : 0.02) but not with vitamin D (*P* : 0.09). We did not find any correlations between calcium or vitamin D and cholesterol, HDL cholesterol or fasting plasma glucose concentrations.

Not only PCOS but also increased body weight had a significant negative effect on serum calcium and 25OHD concentrations. Specifically, obese women in the both groups (PCOS and their controls) had significantly lower vitamin D concentrations than did normal-weight women (*P* = 0.03) (Table 6).

## 4. Discussion

This study determined significantly lower concentrations of calcium, vitamin D, and adiponectin in subjects with PCOS independent to BMI. Furthermore, a potent positive relationship was found between calcium, 25OHD, and adiponectin concentrations. This study revealed that the correlation between lower concentrations of calcium and vitamin D with insulin resistance is dependent to BMI and waist circumference in PCOS. We found that the association of hypoadiponectinemia with PCOS is dependent on vitamin D. To the best of our knowledge, this is the first study in which the possible role of calcium and vitamin D on the hypoadiponectinemia in a sizeable sample of women with PCOS and their controls was investigated.

 Obese women in the both groups had significantly lower calcium and vitamin D concentrations than normal-weight women in this study. These findings are in agreement with previous reports of decreased calcium and vitamin D concentrations in obese, compared with normal-weight patients [5, 6, 19, 20].

 Decreased vitamin D concentrations have been reported in some diseases including type 2 diabetes mellitus (T2DM), obesity, and hypertension [21]. Furthermore, the presence of vitamin D receptors in several tissues such as pancreatic beta cells, adipocyte, and muscle cells suppose, a potent role of this vitamin in the regulation of metabolism of the body. In obese individuals, the impaired vitamin D endocrine system could act on renin-angiotensin system and also in vascular function and induce hypertension [22]. In pancreatic cells, impaired vitamin D actions may contribute to higher intracellular calcium, which makes less insulin secretion. Deterioration of insulin action in peripheral tissues also has been reported in vitamin D deficiency. Some studies reported more important role of PTH than vitamin D in obesity and metabolic syndrome [23]. Although we did not measure PTH in our study, there are some studies reported higher concentrations of PTH in PCOS [24].

In the present study, women with PCOS had significantly lower calcium and 25OHD concentrations than ovulatory normal women. This difference remained significant for both groups after adjustment for BMI. Although lower calcium and 25OHD concentrations in PCOS have been reported previously [25–27], some reports indicated that this reduced levels may result from the presence of obesity and insulin resistance rather than PCOS per se [26, 27]. Regarding the results of the present study, hypovitaminosis D possibly is linked to PCOS and vitamin D supplements may be effective in prevention of PCOS.

Interestingly, we found that hypoadiponectinemia in PCOS is dependent on vitamin D deficiency. Regarding on a confirmed association of low adiponectin concentration in PCOS and its relation to insulin resistance independent of BMI [15], a good explanation for the above findings would be that hypoadiponectinemia may be due to decreased concentrations of vitamin D metabolites.

A significant negative correlation between calcium (and vitamin D with a borderline *P* value of 0.09) with triglyceride concentrations and not cholesterol and plasma glucose was also observed, in agreement with previous reports of a positive association between hypovitaminosis D and metabolic abnormalities in PCOS [28].

Although some data are available for the effect of vitamin D [29] or calcium supplementation [30] on metabolic indexes in obese subjects, there are little intervention trials to evaluate the effect of vitamin D supplementation on metabolic disturbances in PCOS women. One of them has been published in 2009 and showed a significantly increased secretion of the first phase of insulin secretion after treatment with alphacalcidol. A favorable statistically significant change also was observed in the lipid profile [31].

In this study, subjects with PCOS had higher concentrations of DHEA-S than normal subjects. Elevated levels of DHEA-S have been reported in 25% to 50% of women with PCOS. The exact etiology of adrenal androgen excess is not known. Increased adrenocorticotropic hormone (ACTH) production, increased adrenal sensitivity to ACTH, altered steroidogenic enzyme activity (17–20 lyase, 3-beta-hydroxysteroid dehydrogenase activity), and an overproduction of androgens in response to hyperprolactinemia have all been implicated as potential mechanisms [32].

Connection between ovarian estrogen production and adrenal androgen synthesis has been evaluated. Estrogens could have a direct adrenal effect, or their effect could be mediated via prolactin. Estrogens are known to increase pituitary prolactin secretion, which in turn will augment adrenal DHEAS output. The induction of hypoestrogenism with gonadotropin-releasing hormone agonist reduces DHEAS levels. On the other hand, some of these metabolic characteristics may have a genetic background [32].

We did not find any association between vitamin D and androgens or gonadotropin concentrations in our study. These findings suppose no effect of vitamin D or calcium supplementation to reduce hyperandrogenism in PCOS. However, there is one report recently to support the favorable effect of adding calcium and vitamin D to metformin to induce ovulatory cycles in PCOS [33], but larger-scale investigations are definitely needed to clarify this issue.

In conclusion, the results of the present study are in agreement with previous data supporting an association of decreased calcium and vitamin D with PCOS but do not support the independent association of these elements with insulin resistance. 

Moreover, our findings indicate, for the first time, that hypovitaminosis D probably is also linked to PCOS-associated hypoadiponectinemia by means of BMI-independent mechanisms.

 It is very possible that dietary supplementation of these nutrients can reduce risk of subsequent development of diabetes in the subjects with PCOS. The possible beneficiary effect of vitamin D on the prevention of PCOS remains to be revealed.

## Figures and Tables

**Table 1:** Biochemical indexes and clinical characteristics of the women with PCOS and their control group (mean ± SD).

Parameter	PCOS group (*n* : 103)	Control group (*n* : 103)	*P* value
Age (year)	24.8 ± 5.6	26.4 ± 6	0.4
BMI (kg/m^2^)	27.7 ± 5	26.2 ± 5.2	0.9
FPG (mmol/L)	4.6 ± 0.94	4.6 ± 0.5	0.8
Chol (mmol/L)	4.3 ± 0.85	4.2 ± 0.95	0.4
TG (mmol/L)	1.4 ± 0.77	1.18 ± 0.75	0.019
HDL Chol (mmol/L)	1.23 ± 0.3	1.2 ± 0.23	0.2
LH (IU/L)	14.4 ± 12	9.2 ± 8.5	<0.001
FSH (IU/L)	8.1 ± 1.4	9.3 ± 2.9	0.7
LH/FSH	2. 6 ± 2	1.6 ± 1	0.001
Testosterone (nmol/L)	0.023 ± 0.01	0.016 ± 0.01	0.07
DHEA-S (nmol/L)	10.7 ± 11	9.7 ± 10.4	0.02

BMI: body mass index; FPG: fasting plasma glucose; Chol: cholesterol; TG: triglyceride; HDL: high density lipoprotein; FSH: follicle-stimulating hormone; LH: luteinizing hormone.

**Table 2:** Mean (SD) 25OHD, calcium, adiponectin, and insulin concentrations of the women with PCOS and their control group.

Parameter	PCOS group (*n* : 103)	Control group (*n* : 103)	*P* value
Insulin (pmol/L)	80.5 ± 62.5	53.4 ± 29.8	0.004
HOMA index	2.3 ± 1.9	1.5 ± 0.9	0.007
Adiponectin (ng/mL)	8.4 ± 2.7	13.6 ± 5	<0.001
Calcium (mmol/L)	2 ± 0.1	2.55 ± 0.17	<0.001
25OHD (nmol/L)	30 ± 2.99	43.7 ± 5.2	<0.001

HOMA index: homeostasis model assessment index; 25OHD: 25 hydroxy vitamin D.

**Table:** (a)

Dependent variable	Independent variables	Odds ratio	CI (95%)	*P* value
	25OHD	1.6	1.23–1.97	<0.001
	Age	0.9	0.8–1	0.9
PCOS	WC	0.89	0.79–1.01	0.2
	BMI	1.16	0.89–1.43	0.5
	Insulin concentration	1.02	0.89–1.15	0.8

25OHD: 25 hydroxy vitamin D; PCOS: polycystic ovary syndrome; WC: waist circumference; BMI: body mass index.

**Table:** (b)

Dependent variable	Independent variables	Odds ratio	CI (95%)	*P* value
PCOS	Calcium	4.2	3.1–5.3	<0.001
	Adiponectin	2.3	1.88–2.7	0.04
	Age	1.01	0.91–1.11	0.8
	WC	1	0.92–1.08	0.9
	BMI	0.87	0.62–1.12	0.6
	Insulin concentration	1.04	0.98–1.08	0.3

PCOS: polycystic ovary syndrome; WC: waist circumference; BMI: body mass index.

**Table 4:** Logistic regression analyses of adiponectin to predict PCOS after adjustment for 25OHD.

Dependent variable	Independent variables	Odds ratio	CI (95%)	*P* value
	Adiponectin	0.7	0.33–1.07	0.06
	Age	0.9	0.8–1.1	0.9
PCOS	WC	0.86	0.74–1.01	0.2
	BMI	1.4	1–1.8	0.3
	25OHD	1.9	1.3–2.5	0.002

25OHD: 25 Hydroxy vitamin D; PCOS: Poly Cystic Ovary Syndrome; WC: Waist circumference; BMI: Body Mass Index.

**Table 5:** Multiple linear regression analyses of association between insulin concentration and 25OHD in subjects with PCOS.

Dependent Variable	Independent Variables	Odds ratio	CI (95%)	*P*-value
25OHD	Insulin	1.1	0.95–1.05	0.2
	Age	1.8	0.8–1.1	0.06
	WC	1.3	0.98–1.15	0.1
	BMI	1.3	0.99–1.5	0.9

25OHD: 25 hydroxy vitamin D; PCOS: polycystic ovary syndrome; WC: waist circumference; BMI: body mass index.

**Table 6:** Differences in calcium and 25OHD in the subjects with and without PCOS based on their BMI.

Parameter	PCOS group (*n* : 103)				Control group (*n* : 103)			
	Normal weight (*n* = 34)	Overweight (*n* = 50)	Obese (*n* = 19)	*P*	Normal weight (*n* = 33)	Overweight (*n* = 54)	Obese (*n* = 16)	*P*
Calcium (mmol/L)	2.07 ± 0.1	2.02 ± 0.17	1.9 ± 0.1	0.2	2.6 ± 0.15	2.5 ± 0.15	2.37 ± 0.15	0.003
25OHD (nmol/L)	31.2 ± 2.49	30.9 ± 4.7	28.9 ± 2.9	0.03	43.7 ± 3.7	43.9 ± 5.2	39.4 ± 2.9	0.02
